# Cross-Vendor Test-Retest Validation of Diffusion Tensor Image Analysis along the Perivascular Space (DTI-ALPS) for Evaluating Glymphatic System Function

**DOI:** 10.14336/AD.2023.0321-2

**Published:** 2024-08-01

**Authors:** Xiaodan Liu, Giuseppe Barisano, Xingfeng Shao, Kay Jann, John M Ringman, Hanzhang Lu, Konstantinos Arfanakis, Arvind Caprihan, Charles DeCarli, Brian T Gold, Pauline Maillard, Claudia L Satizabal, Elyas Fadaee, Mohamad Habes, Lara Stables, Herpreet Singh, Bruce Fischl, Andre van der Kouwe, Kristin Schwab, Karl G Helmer, Steven M Greenberg, Danny J.J Wang, for the MarkVCID Consortium

**Affiliations:** ^1^Laboratory of FMRI Technology (LOFT), Mark & Mary Stevens Neuroimaging and Informatics Institute, University of Southern California, Los Angeles, CA, USA.; ^2^Zilkha Neurogenetic Institute, University of Southern California, Los Angeles, CA, USA.; ^3^Department of Radiology and Biomedical Imaging, University of California, San Francisco, San Francisco, CA, USA.; ^4^Department of Neurosurgery, Stanford University, Stanford, CA, USA.; ^5^Department of Neurology, Keck School of Medicine, University of Southern California, Los Angeles, CA, USA.; ^6^Department of Radiology, Johns Hopkins University School of Medicine, Baltimore, MD, USA.; ^7^Department of Biomedical Engineering, Illinois Institute of Technology, Chicago, IL, USA.; ^8^Department of Diagnostic Radiology and Nuclear Medicine, Rush University, Chicago, IL, USA.; ^9^Rush Alzheimer’s Disease Center, Rush University Medical Center, Chicago, IL, USA.; ^10^The Mind Research Network, Albuquerque, NM, USA.; ^11^Department of Neurology, University of California, Davis, Davis, CA, USA.; ^12^Department of Neuroscience, University of Kentucky, Lexington, KY, USA.; ^13^Population Health Sciences and Glenn Biggs Institute for Neurodegenerative Diseases, University of Texas Health Science Center at San Antonio, San Antonio, TX, USA.; ^14^Neuroimage Analytics Laboratory and Biggs Institute Neuroimaging Core, Glenn Biggs Institute for Neurodegenerative Diseases, University of Texas Health Science Center at San Antonio, San Antonio, TX, USA.; ^15^Department of Neurology, University of California, San Francisco, San Francisco, CA, USA.; ^16^Department of Neurology, Massachusetts General Hospital, Boston, MA, USA.; ^17^Department of Radiology, Harvard Medical School, Boston, MA, USA.; ^18^Athinoula A. Martinos Center for Biomedical Imaging, Massachusetts General Hospital, Charlestown, MA, USA.; ^19^Division of Health Sciences and Technology, Massachusetts Institute of Technology, Computer Science and AI Lab, Cambridge, Massachusetts, USA.

**Keywords:** Diffusion tensor image analysis along the perivascular space (DTI-ALPS), Neurofluids, Brain, glymphatic system, MarkVCID consortium

## Abstract

The diffusion tensor image analysis along the perivascular space (DTI-ALPS) method was proposed to evaluate glymphatic system (GS) function. However, few studies have validated its reliability and reproducibility. Fifty participants’ DTI data from the MarkVCID consortium were included in this study. Two pipelines by using DSI studio and FSL software were developed for data processing and ALPS index calculation. The ALPS index was obtained by the average of bilateral ALPS index and was used for testing the cross-vendor, inter-rater and test-retest reliability by using R studio software. The ALPS index demonstrated favorable inter-scanner reproducibility (ICC=0.77 to 0.95, *P*< 0.001), inter-rater reliability (ICC=0.96 to 1, *P*< 0.001) and test-retest repeatability (ICC=0.89 to 0.95, *P*< 0.001), offering a potential biomarker for in vivo evaluation of GS function.

## INTRODUCTION

The glymphatic system (GS) is a recently discovered brain-wide perivascular fluid transport system in the cerebral nervous system (CNS). This system was thought to clear interstitial fluid (ISF) of waste products from the brain via the ISF-cerebrospinal fluid (CSF) exchange facilitated by the aquaporin-4 (AQP4) water channels expressed at the vascular endfeet of astrocytes [1]. The GS serves as the brain’s “front end” drainage pathway, which is connected to a downstream lymphatic network via meningeal lymphatics, cranial nerves, and large vessels for removing the waste and excess fluid from the CNS [1, 2]. Therefore, the GS is essential for maintaining cerebral fluid homeostasis across the lifespan. A growing number of studies have demonstrated that the impairment of glymphatic transport was associated with several neurological diseases, including cerebral small vessel disease (cSVD) [3-6], Alzheimer’s disease (AD) [7, 8], hydrocephalus [7, 9], diabetes [10, 11], traumatic brain injury [12, 13] and stroke [14, 15]. Additionally, GS dysfunction is related to sleep disorder as well as tau and beta-amyloid (Aβ) protein accumulations, which underlie the pathogenesis of cognitive impairment and dementia [16-21]. The utility of magnetic resonance imaging (MRI) for in vivo investigation of the GS has recently gained momentum. Dynamic contrast enhanced (DCE) MRI and T1 mapping MRI techniques allows the visualization of glymphatic flow pathways in vivo and further modeling the glymphatic transport process, providing insights into the GS function [5, 6, 9, 22, 23]. However, these methods require intrathecal administration of contrast medium, which is not suitable for humans.

In recent years, a novel method named “diffusion tensor image analysis along the perivascular space (DTI-ALPS)” was proposed by using diffusion MRI for non-invasive evaluation of the clearance function of the GS [24]. In this method, the motion of water molecules in the direction of the perivascular space was assessed by measuring diffusivity. The perivascular space was hypothesized to run the same direction as the medullary veins at the level of the lateral ventricle body that run perpendicular to the ventricle wall. This right-left direction was defined as x-axis. On the plane of this area, the adjacent projection fibers run in the head-foot direction and association fibers run in the anterior-posterior direction, which are orthogonal to the direction of perivascular space and defined as y-axis and z-axis respectively [24]. When there’s histological changes along the direction of perivascular space, it will affect diffusivity of both projection and association fibers. The ALPS index is therefore defined by the ratio of the mean of x-axis diffusivity in the area of projection fibers (D_xxproj_) and x-axis diffusivity in the area of association fibers (D_xxassoc_) to the mean of the y-axis diffusivity in the area of projection fibers (D_yyproj_) and z-axis diffusivity in the area of association fibers (D_zzassoc_) [24].

Nowadays, there have been nearly 40 clinical studies investigating GS function by using the DTI-ALPS method, covering a range of neurological disorders, such as normal aging, cSVD, stroke, dementia, TBI, hydrocephalus, epilepsy, multiple sclerosis, sleep disorder and peritumoral edema [24-34]. In particular, the ALPS index was shown to correlate with the conventional DCE MRI method using intrathecal injection of Gd-based contrast agent, conventional MRI biomarkers of cSVD, as well as the clinical assessments of cognitive impairment [32, 35]. However, studies on the cross-vendor and test-retest reliability of DTI-ALPS method are lacking. Only one recent single center study performed evaluation on the reproducibility of ALPS index [36]. The current study aimed to perform cross-vendor, inter-rater and test-retest validations of DTI-ALPS method by using a cohort from the MarkVCID consortium [37].

## MATERIALS AND METHODS

The MRI data used in this study were acquired as part of the MarkVCID consortium. The phase I of MarkVCID consortium consisted of seven sites: Johns Hopkins University School of Medicine (JHU); Rush University Medical Center/Illinois Institute of Technology (RUSH); University of California San Francisco, Davis and Los Angeles (UCSF/UCD/UCLA); University of Kentucky (UKY); University of New Mexico Health Science Center (UNM); University of Southern California (USC) and the University of Texas Health Science Center at San Antonio (UTHSCSA, operating as part of the Cohorts for Heart and Aging Research in Genomic Epidemiology [CHARGE] consortium stie); and a central coordinating center (Massachusetts General Hospital) working with the National Institute of Neurological Disorders and Stroke (NINDS) and the National Institute on Aging (NIA) under cooperative agreements. The instrumental validation included: (1) inter-scanner reproducibility (differences across different MRI scanners from different sites in the same group of individuals within an interval of 3 to 90 days), (2) inter-rater reliability (differences between two raters analyzing the same MRI dataset), (3) test-retest repeatability (differences between two scans obtained for the same individual and MRI scanner with an interval of 1 to 14 days). 3T MRI scanners used by the seven sites included two Siemens systems (TIM Trio and Prisma), one Philips system (Achieva) and one General Electric (GE) system (750W). The participants included in this study were in the age range of 54 to 89 years (71± 9 years, 14 males, 36 females) Participants with unstable major medical illness, major primary psychiatric disorder, prevalent stroke at the MRI assessment or other neurological disorders that might confound the diffusivity analysis were excluded. The participants had Fazekas periventricular white matter (PVWM) scores of 0 to 3 (2.0±0.8), Fazekas deep white matter (DWM) scores of 0 to 3 (1.8±1.0), Clinical Dementia Rating scale (CDR) scores of 0 to 1 (0.6 ±1.0) and Montreal Cognitive Assessment (MoCA) of 10 to 30 (25 ±4). The institutional review boards at all participating institutions approved this study and subjects or their legal representative gave written informed consent.

**Figure 1.**
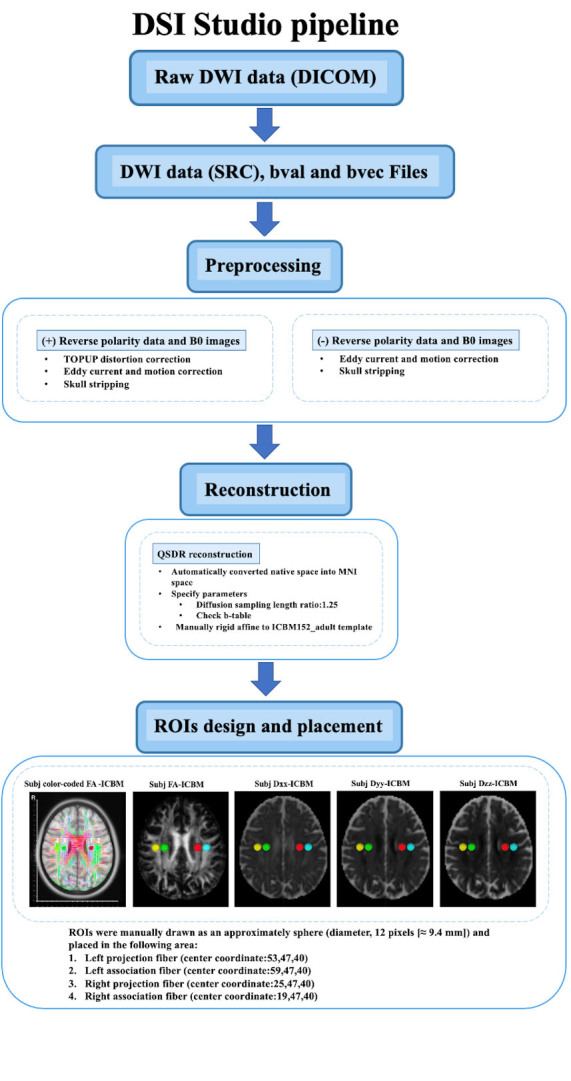
The DTI data processing workflow of DSI Studio pipeline.

### MRI acquisition

Diffusion tensor imaging (DTI) acquisition protocol for MarkVCID used a single shell, b=1000s/mm^2^, 40-direction with a voxel size of 2.0x2.0x2.0 mm^3^ and six b=0 s/mm^2^. The phase-encoding (PE) direction for the 40-direction dataset was chosen to be “PA” and a separate, shorter (two b=0 scans, six b=1000 scans) in which the PE direction was reversed to “AP”. The reverse PE polarity data were used to estimate and correct image distortions. Imaging parameters for MarkVCID sequences on different MRI scanners (Philips Achieva, Siemens Prisma and Trio, GE 750W) have been previously described [38], and are summarized here: Field of View (FOV) = 256x256 mm^2^, matrix = 128x128, resolution=2x2 mm^2^, slice thickness = 2mm, 80 slices, Repetition Time (TR)/ Echo Time (TE) = 9014/75ms (Philips Achieva), 8600/68 ms (Siemens Prisma), 9800/84ms (Siemens Trio), 14453/75 ms (GE 750W), b=0 and 1000 s/mm^2^ with 40 directions.

### DTI data processing protocol

**Figure 2.**
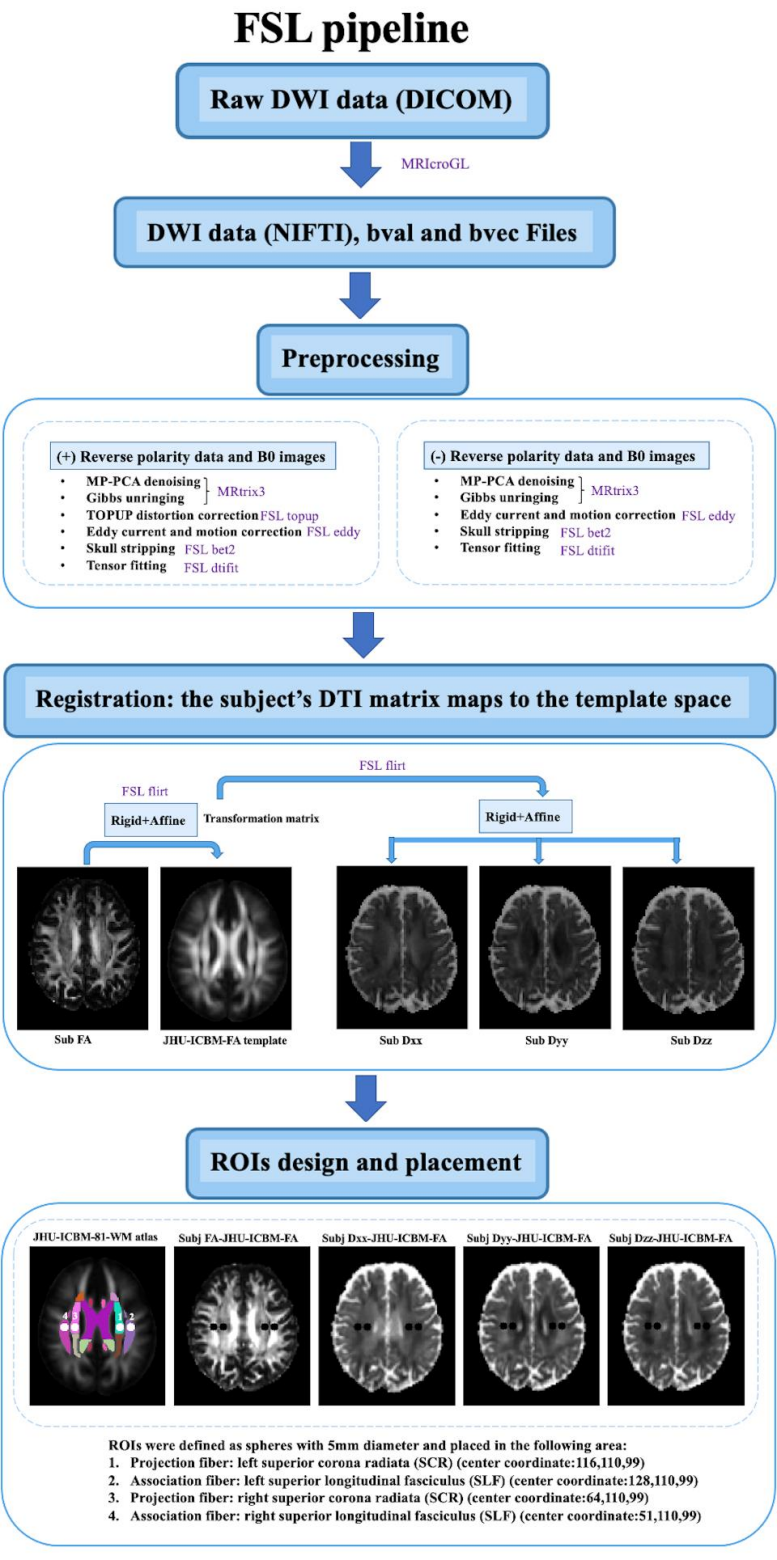
The DTI data processing workflow of FSL pipeline.

The general DTI data processing includes: (1) the DTI images were corrected for phase distortion with the reverse PE volumes, followed by eddy current and head movement (2) the color-coded fractional anisotropy (FA) map and diffusive maps in the direction of x-axis (right-left; D_xx_), y-axis (anterior-posterior; D_yy_) and z-axis (inferior-superior; D_zz_) were generated, (3) the reconstructed image or FA map was transformed into the template space using both linear and nonlinear transformations, and the transformation matrix was applied to all diffusive maps, (4) regions of interest (ROIs) were placed in the areas of projection and association fibers at the level of the lateral ventricle body. The DTI data which lacked the reverse PE data or didn’t have the b=0 image skipped the TOPUP correction. We implemented these processing steps using DSI Studio graphic-user interface software version 10.15 (DSI Studio GUI; https://dsi-studio.labsolver.org/) (Fig. 1) and FMRIB software Library version 6.0 (FSL; Oxford Centre for Functional MRI of the Brain, Oxford, UK; https://fsl.fmrib.ox.ac.uk/fsl/) (Fig. 2). The pipelines are described below.

### DSI Studio pipeline

In this pipeline, all the processing steps were performed with DSI Studio GUI. The 4D DTI volume DICOM files were firstly converted to SRC files, then the images were preprocessed by using TOPUP/EDDY and motion program for phase distortion, eddy current and motion corrections, reconstructed by using Q-Space Diffeomorphic reconstruction (QSDR) method, which is the MNI version of generalized Q-sampling imaging (GQI) that can automatically transform the images into the MNI space and normalized to the ICBM152_adult template. Subsequently, the color-coded FA maps and x-, y- and z-axis diffusivity maps were estimated and outputted. The projection and association fibers were identified on the color-coded FA maps, and the spherical ROIs were manually drawn (diameter, 12 pixels [≈ 9.4 mm]) and placed in the areas of bilateral projection and association fibers at the level of lateral ventricle body. The center coordinates of ROIs were as follows: left projection fiber (53,47,40), left association fiber (59,47,40), right projection fiber (25,47,40), right association fiber (19,47,40). The total 4 ROIs were placed onto the color-coded FA and diffusivity maps of each subject respectively. Then the diffusivity values of D_xx_, D_yy_ and D_zz_ of bilateral projection and association fibers were obtained for the ALPS calculation (Fig. 1).

## FSL pipeline

The 4D DTI volume DICOM files were converted to NIFTI files by using MRIcroGL GUI. We created an in-house bash script to compute the ALPS index using the DTI images as input and including FSL and MRtrix3 commands. The DTI images underwent artifact corrections using Marchenko-Pastur Principal Component (MP-PCA) denoising algorithm and Gibbs-unringing using MRtrix3 command line “*dwisenoise*” and “*mrdegibbs*”, Corrections of susceptibility-induced distortions, eddy currents and movements were accomplished with FSL command line “*topup*” and “*eddy*”. The FA map and x-, y- and z-axis diffusivity maps were generated using FSL command line “*dtifit*”. The FA map of each subject was co-registered to the JHU-ICBM-FA template and the transformation matrix was applied to all the diffusivity maps by using FSL command line “*flirt*”. The projection and association fibers at the level of lateral ventricle body were recognized as the superior corona radiata (SCR) and the superior longitudinal fasciculus (SLF) based on the JHU-ICBM-DTI-81-white-matter Labeled Atlas and the ROIs were automatically defined as spheres with 5mm diameter in the areas of bilateral SCR and SLF which applied on all subjects’ diffusivity maps. The center coordinates of ROIs were as follows: left SCR (116,110,99), left SLF (128,110,99), right SCR (64,110,99) and right SLF (51,110,99) JHU-ICBM-FA template. The diffusivity values of D_xx_, D_yy_ and D_zz_ of bilateral SLF and SCR were automatically outputted for the ALPS calculation (Fig. 2).

### ALPS index calculation

The ALPS index is defined by the average of bilateral ALPS indexes (mean ALPS index), which is by the ratio of the mean of x-axis diffusivity in the area of projection fibers (D_xxproj_) and x-axis diffusivity in the area of association fibers (D_xxassoc_) to the mean of the y-axis diffusivity in the area of projection fibers (D_yyproj_) and z-axis diffusivity in the area of association fibers (D_zzassoc_)[24] as follows:

$$\mathrm{ALPS index}=\frac{\mathrm{mean}(D\mathrm{xxproj},D\mathrm{xxassoc})}{\mathrm{mean}(D\mathrm{yyproj},D\mathrm{zzassoc})}$$

The ALPS index was used for the reliability and reproducibility validations of the DSI Studio and FSL pipelines respectively.

### Statistical analysis

Inter-scanner reproducibility: Inter-scanner re-producibility of ALPS index among the four scanners was evaluated by using consistency interclass correlation coefficient (ICC)[39]. It uses a two-way random effects model with single measures and consistency form, which is noted as ICCc and calculated as follows:

$$\mathrm{ICCc}=\frac{\mathrm{MS}R-\mathrm{MS}E}{\mathrm{MS}R+(k-1)\mathrm{MS}E}$$

where MS_R_ = the mean square for rows (i.e., participants), MS_E_ = the mean square error, k = the number of raters. Pairwise ICCc between each pair of scanners were also computed by using R studio software (version 2022.07.02 + 576.pro12), and the significant level was defined as *P*<0.05.

Inter-rater reliability: To evaluate inter-rater reliability of ALPS index between two raters who independently analyzed the same DTI dataset, we computed the ICC using a two-way random effects model with single measure and absolute agreement form, is noted as ICC_AA_ and calculated as follows:

$$\mathrm{ICC}\mathrm{AA}=\frac{\mathrm{MS}R-\mathrm{MS}E}{\mathrm{MS}R+\left(k-1\right)\mathrm{MS}E+\frac{k}{n}(\mathrm{MS}C-\mathrm{MS}E)}$$

where MS_c_ =the mean square for columns (i.e., raters), n = the number of subjects. ICC_AA_ estimates agreement between measures without allowing systematic error. Pairwise ICC_AA_ between raters were also computed by using R studio, and the significant level was defined as *P*<0.05.

Test-retest repeatability: To evaluate test-retest repeatability of ALPS index, we computed ICC_AA_ between two scanning sessions obtained for the same individual and MRI scanner within 14 days by using a two-way random-effects model with single measure and absolute agreement form as described above. Pairwise ICC_AA_ between test and retest were also computed by using R studio, and the significant level was defined as *P*<0.05.

**Figure 3.**
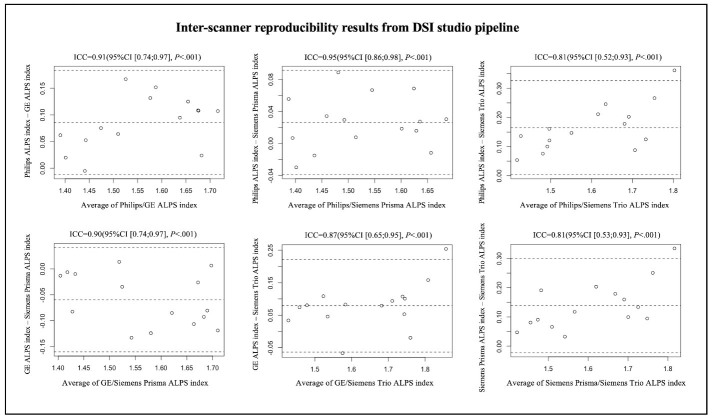
Bland-Altman plots of the inter-scanner reproducibility results from DSI Studio pipeline.

**Figure 4.**
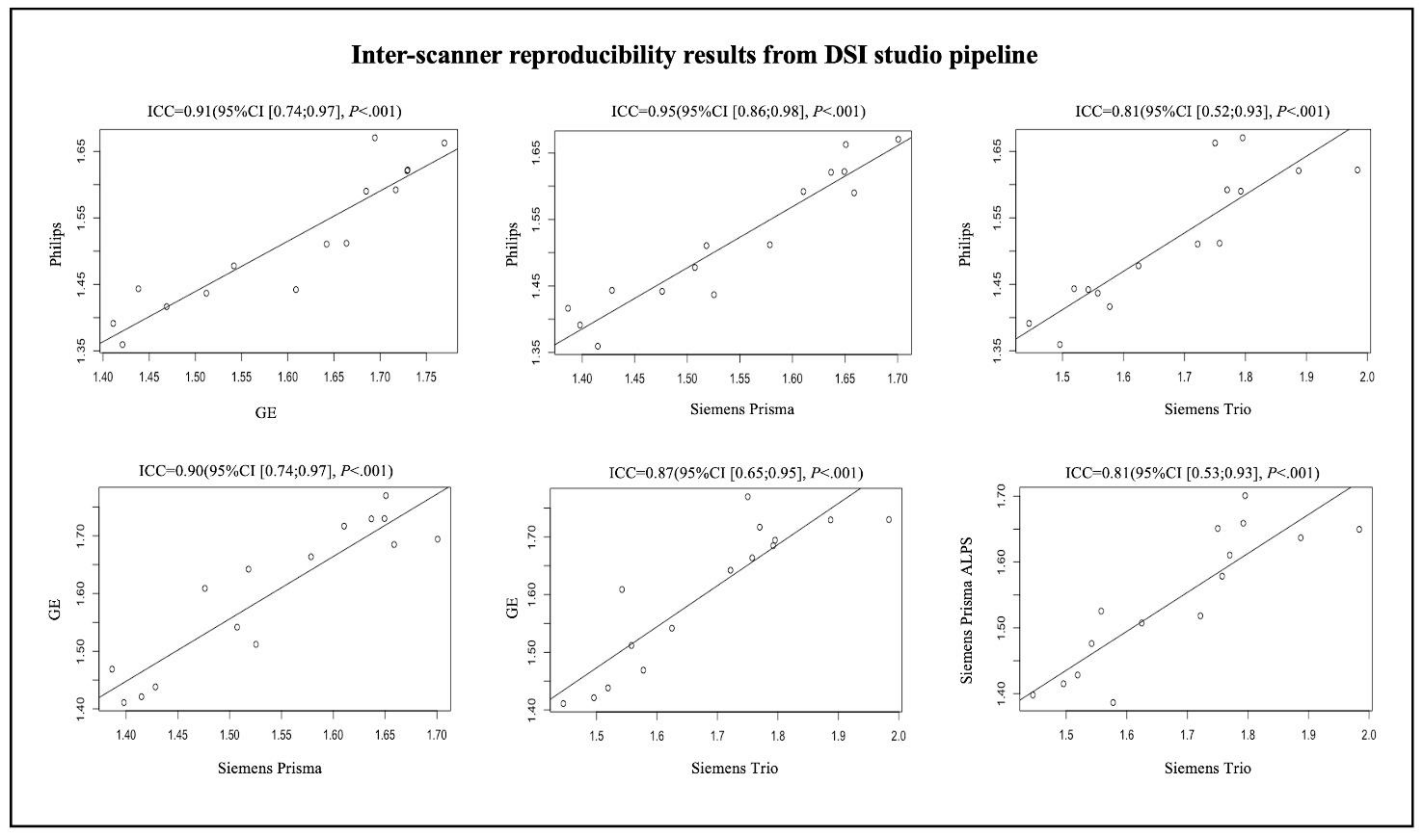
Scatterplots of the inter-scanner reproducibility results from DSI Studio pipeline.

For visualization of the results from the analyzes of inter-scanner reproducibility, inter-rater reliability and test-retest repeatability, Bland-Altman plot was created by using R studio. The terms poor, moderate, good and excellent were defined as ICC values < 0.5, between 0,5 and 0.75, between 0.75 and 0.9, and > 0.9, respectively [39].

**Table 1:** Intra-class coefficients of mean ALPS index between scanners, raters, and test-retest sessions.

Validation items		DSI Studio pipeline	FSL pipeline
**Inter-scanner reproducibility** **(N=15)**	**Philips-GE**	0.91(95%CI [0.74;0.97], *P*<0.001)	0.84(95%CI [0.58;0.94], *P*<0.001)
	**Philips-Siemens_Prisma**	0.95(95%CI [0.86;0.98], *P*<0.001)	0.93(95%CI [0.80;0.98], *P*<0.001)
	**Philips-Siemens_Trio**	0.81(95%CI [0.52;0.93], *P*<0.001)	0.80(95%CI [0.51;0.93], *P*<0.001)
	**GE-Siemens_Prisma**	0.90(95%CI [0.74;0.97], *P*<0.001)	0.84(95%CI [0.60;0.94], *P*<0.001)
	**GE-Siemens_Trio**	0.87(95%CI [0.65;0.95], *P*<0.001)	0.77(95%CI [0.44;0.92], *P*<0.001)
	**Siemens_Prisma-Siemens_Trio**	0.81(95%CI [0.53;0.93], *P*<0.001)	0.81(95%CI [0.51;0.93], *P*<0.001)
**Inter-rater reliability (N=15)**		0.96(95%CI [0.94;0.98], *P*<0.001)	1(95%CI [1;1], *P*<0.001)
**Test-retest repeatability (N=35)**		0.89(95%CI [0.79;0.94], *P*<0.001)	0.95(95%CI [0.90;0.97], *P*<0.001)

## RESULTS

### Inter-scanner reproducibility

**Figure 5.**
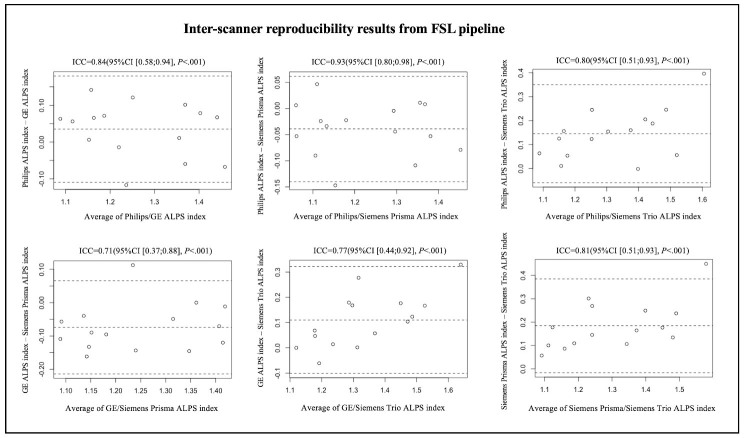
Bland-Altman plots of the inter-scanner reproducibility results from FSL pipeline.

**Figure 6.**
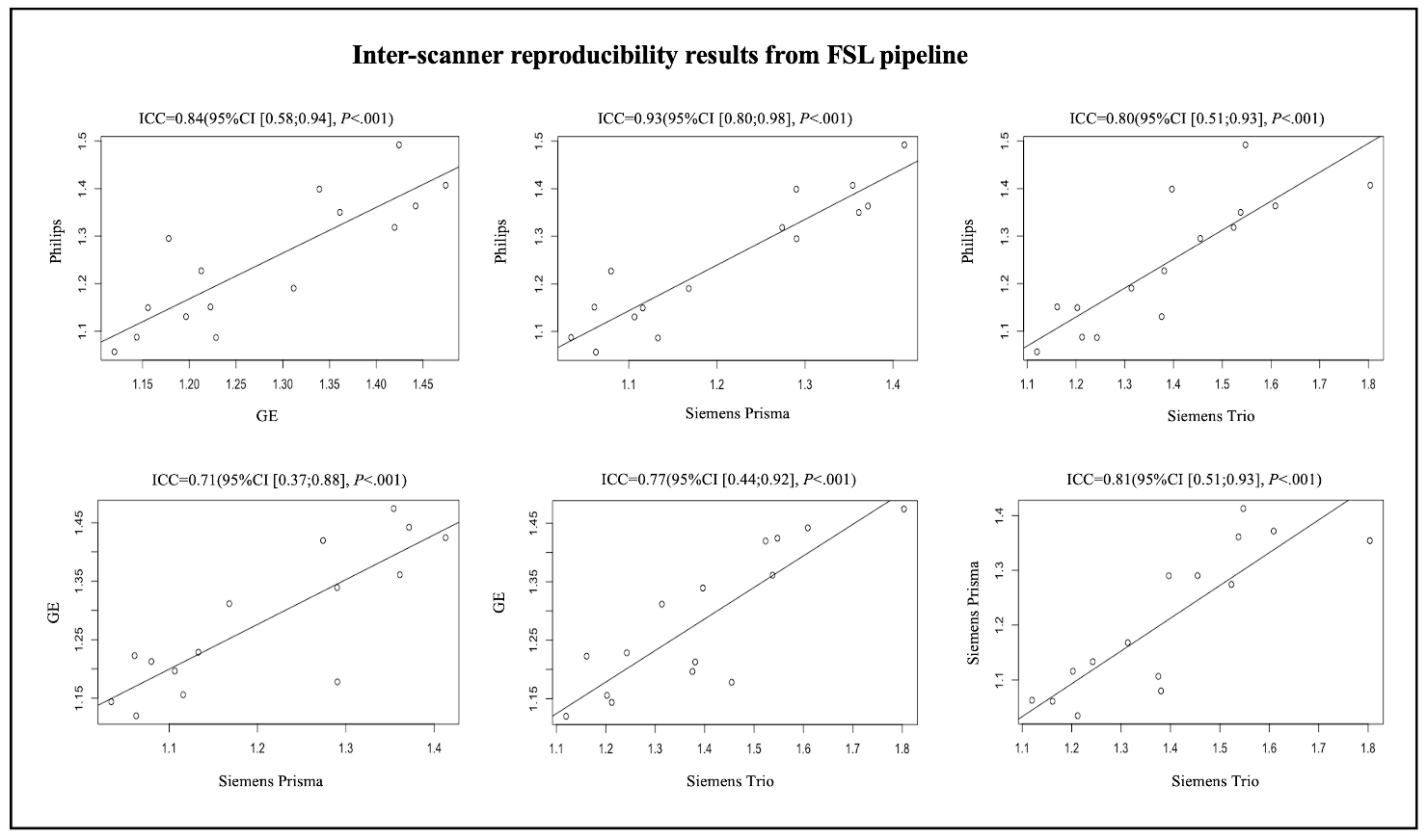
Scatterplots of the inter-scanner reproducibility results from FSL pipeline.

A total of 15 participants were recruited for the inter-scanner study, each participant was scanned on four MarkVCID sites’ MRI scanners, including Philips Achieva, Siemens Trio, Siemens Prisma and GE750W, resulting in a final sample size of 60 ALPS index measures (4 scanners × 15 subjects). The Bland-Altman plots and scatterplots illustrate the results of ICCc between each pair of scanners for 15 participants’ ALPS index, which were analyzed by using DSI Studio pipeline and FSL pipeline respectively (Fig. 3, Fig. 4, Fig. 5 and Fig. 6). Pairwise ICCc were all significant (*P*<0.001): 0.91 (95%CI [0.74;0.97]) (DSI studio pipeline) and 0.84 (95%CI [0.58;0.94]) (FSL pipeline) for Philips-GE, 0.95 (95%CI [0.86;0.98]) (DSI studio pipeline) and 0.93 (95%CI [0.80;0.98]) (FSL pipeline) for Philips-Siemens_Prisma, 0.81 (95%CI [0.52;0.93]) (DSI studio pipeline) and 0.80 (95%CI [0.51;0.93]) (FSL pipeline) for Philips-Siemens_Trio, 0.90 (95%CI [0.74;0.97]) (DSI studio pipeline) and 0.84 (95%CI [0.60;0.94]) (FSL pipeline) for GE-Siemens_Prisma, 0.87 (95%CI [0.65;0.95] (DSI studio pipeline) and 0.77 (95%CI [0.44;0.92]) (FSL pipeline) for GE-Siemens_Trio, 0.81(95%CI [0.53;0.93]) (DSI studio pipeline) and 0.81 (95%CI [0.51;0.93]) (FSL pipeline) for Siemens_Prisma-Siemens_Trio, respectively (See Table 1).

### Inter-rater reliability

**Figure 7.**
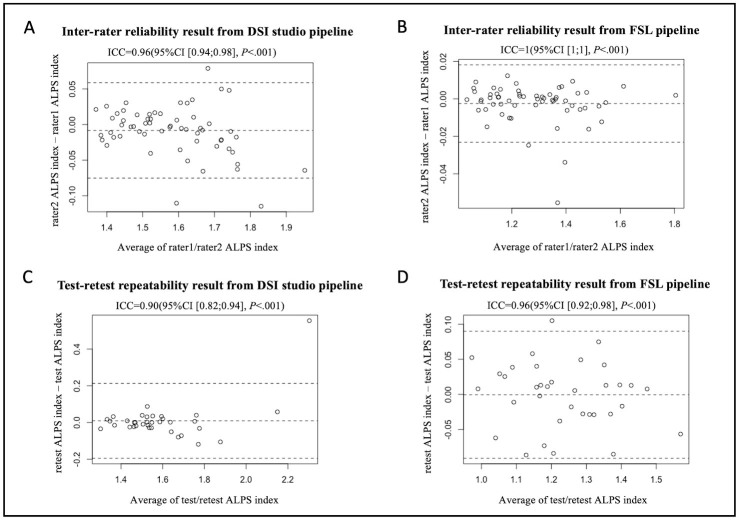
**Bland-Altman plots of the inter-rater reliability and test-retest repeatability results**. (A and B) The inter-rater reliability results from DSI Studio pipeline and FSL pipeline. (C and D) The test-retest repeatability results from DSI Studio pipeline and FSL pipeline.

**Figure 8.**
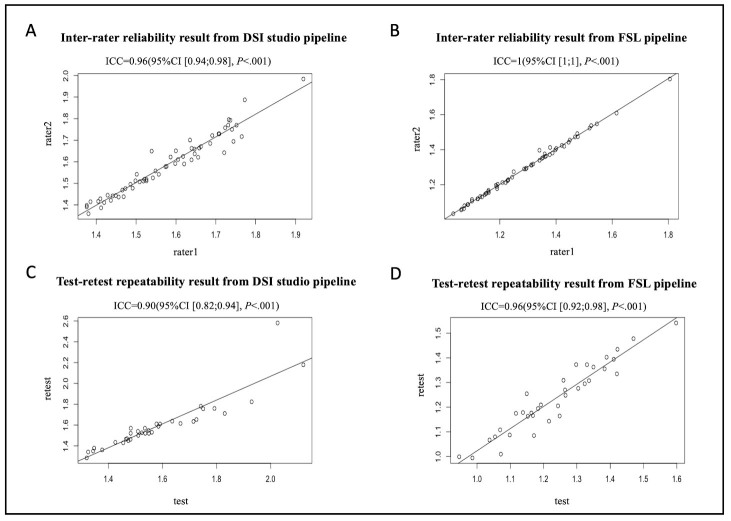
**Scatterplots of the inter-rater reliability and test-retest repeatability results**. (A and B) The inter-rater reliability results from DSI Studio pipeline and FSL pipeline. (C and D) The test-retest repeatability results from DSI Studio pipeline and FSL pipeline.

The 15 subjects in the inter-scanner study were also used for the inter-rater reliability study. Two raters (X.L. and G.B. with 10- and 7-years’ experience analyzing diffusion MRI data respectively) analyzed the 15 subjects’ DTI images from four MRI scanners (Philips Achieva, Siemens Trio, Siemens Prisma and GE750W) by using DSI Studio and FSL pipeline for calculating the ALPS index independently. The Bland-Altman plots and scatterplots illustrate the results of ICC_AA_ between raters for 15 participants’ ALPS index, which were analyzed by using DSI Studio and FSL pipeline respectively (Fig. 7A, Fig. 7B, Fig. 8A and Fig. 8B). The overall pairwise ICC_AA_ between raters were 0.96 (95%CI [0.94;0.98]) (DSI studio pipeline) and 1(95%CI [1;1]) (FSL pipeline) (*P*<0.001) (See Table 1).

**Table 2:** Differences between DSI Studio pipeline and FSL pipeline.

	DSI Studio pipeline	FSL pipeline
**Denoising**	No	MP-PCA denoising (MRtrix3)
**Gibbs ringing artifact removing**	No	Gibbs-unringing (MRtrix3)
**Template for co-registration**	ICBM152_adult	JHU-ICBM-FA-1mm
**Registration method**	Automatically rigid and affine transformation Manually adjustment was allowed	Automatically rigid and affine transformation
**ROIs design and placement**	ROIs were drawn manually as an approximately spheres (diameter, 12 pixels [≈ 9.4 mm]) and placed in the areas of bilateral projection and association fibers onto the individual subject’s color-coded FA-ICBM map transformed to the ICBM152_adult template	ROIs were automatically defined as spheres with 5mm diameter and placed in the areas of bilateral superior corona radiata (SCR)(projection fiber) and superior longitudinal fasciculus (SLF)(association fiber) onto the JHU-ICBM-FA template

### Test-retest repeatability

A total of 35 participants who underwent two sessions of MR scans within 14 days at 7 participating sites of the MarkVCID consortium were included for the test-retest repeatability. The Bland-Altman plots and scatterplots illustrate the results of ICC_AA_ between test and retest for 15 participants’ ALPS index, which were analyzed by using DSI Studio and FSL pipeline respectively (Fig. 7C, Fig. 7D, Fig. 8C and Fig. 8D). The overall pairwise ICC_AA_ between test and retest were 0.89 (95%CI [0.79;0.94]) (DSI Studio pipeline) and 0.95 (95%CI [0.90;0.97]) (FSL pipeline) (*P*<.001) (See Table 1).

## DISCUSSION

To our knowledge, this is the first multicenter, cross-vendor test-retest validation study of DTI-ALPS method. We performed this study on DTI datasets from the MarkVCID consortium and reported the instrumental validation results, including inter-scanner reproducibility, inter-rater reliability, and test-retest repeatability using two analysis pipelines. The DTI-ALPS analysis pipelines are expected to be implemented in future multicenter clinical validation studies for a novel imaging biomarker for evaluating GS clearance function in neurological disorders in particular cSVD. In the present study, we used ALPS index for the instrumental validation and found ALPS index had good consistencies across four scanners (Philips Achieva, Siemens Trio, Siemens Prisma and GE750W), excellent inter-rater reliability and high agreement between test and retest sessions. In previous DTI-ALPS studies, left side ALPS index was commonly used, considering that the recruited subjects were right-handed, and the fiber tracts are thick enough to place ROIs. The ALPS index is calculated by the average of bilateral ALPS indexes, which was firstly proposed by Zhang W, et al. [32] and applied to the investigation of the glymphatic clearance function in cSVD patients. The authors reported that the ALPS index had excellent inter- and intra-observer reliability (ICC = 0.930 and 0.937)[32]. Additionally, they detected that the ALPS index was significantly related to the glymphatic clearance function evaluated on DCE MRI with intrathecal gadolinium-based contrast administration and was correlated with the MRI biomarkers of cSVD, including white matter hyperintensities (WMHs), numbers of lacunas and microbleeds and enlarged perivascular spaces (ePVS). Another study validated the correlation of ALPS index, left ALPS index and right ALPS index to the demographics and vascular risk factors by using multiple regression model in normal aging subjects. The authors found that the ALPS index was correlated with demographics and vascular risk factors, including age, sex, hypertension, and DMV scores when compared to left ALPS index or right ALPS index [40]. However, the ALPS index, left ALPS index and right ALPS index were not found to have significant associations with cSVD imaging markers in this study [40].

Despite that the DTI-ALPS method had been extensively used to investigate the glymphatic clearance in a variety of pathologies, there has been a lack of studies to evaluate the reliability and reproducibility of DTI-ALPS method. Recently, Taoka T et al.,[36] conducted a study named “Changes in Alps index on Multiple conditiON acqulsition eXperiment (CHAMONIX)”, which was a single center study that evaluated the effect of various MRI scanning parameters on the ALPS calculation. In addition, the inter-scanner (two 3T MRI scanners namely Vantage Centurian and Siemens Magnetom Prisma) reliability and test-retest repeatability (four times repeated scans within 1 week) were evaluated. The authors found similar good inter-scanner reproducibility (ICC range 0.775-0.828) and test-retest repeatability (ICC = 0.87) of ALPS index. The authors also found that the alternations in the imaging plane, head position, and scanning parameters, i.e. TR/TE largely influenced the reproducibility of the ALPS index. In the present study, we noticed the relatively lower inter-scanner consistency between the Siemens_Trio scanner and other three scanners (Philips Achieva, Siemens Prisma and GE750W). There may be two reasons: 1) Some of DTI data acquired on Siemens_Trio scanner lacked the reverse PE image and b=0 image, therefore the TOPUP distortion correction could not be performed. 2) The TE on Siemens Trio (TE=84ms) was longer than those on other scanners, which may affect the reliability of DTI measurements, as suggested by CHAMONIX study [36]. Our result suggests that the DTI data acquisition and processing step is also one of the factors that may affect the ALPS calculation.

In the present study, we applied two DTI data processing pipelines: DSI Studio and FSL pipeline, which are commonly used in existing DTI-ALPS studies. There’re several differences between DSI studio and FSL pipelines (See Table 2). Firstly, DSI Studio provided a user-friendly graphic interface which is able to perform the TOPUP distortion, eddy current and motion corrections, linear and non-linear normalization from native space to a standard space (e.g., MNI) and reconstructions for estimating various DTI metrics, while the FSL pipeline consisted of an in-house bash script based on the FSL commands, which can be executed as a batch job for parallel processing of large datasets. Secondly, additional artifact corrections, including MP-PCA denoising and Gibbs unringing via MRtrix3 commands were included in the FSL pipeline. MP-PCA reduces the signal fluctuations induced by the motion of electrons or ions (i.e., the thermal noise)[41].

Gibbs unringing reduces Gibbs-ringing artifact which is typically observed near sharp edges and improves the accuracy of estimating DTI metrics [42]. MP-PCA denoising and Gibbs unringing have been recommended to be the first steps of the DTI processing pipeline [41, 43] and has been involved in the processing pipeline in previous DTI-ALPS studies [40, 44]. Thirdly, in DSI Studio pipeline, the ROIs were manually drawn (diameter, 12 pixels [≈ 9.4 mm]) and placed in the areas of bilateral projection and association fibers on the individual subject’s color-coded FA map transformed into the ICBM152_adult template, while in FSL pipeline, the ROIs were defined as spheres with 5mm diameter and placed in the area of bilateral projection fibers (SCR) and association fibers (SLF) on JHU-ICBM-FA template. The SCR and SLF were identified based on the JHU-ICBM-DTI-81-white-matter Labeled atlas. Fourthly, DSI studio allows the user to manually correct the automatic registration of the individual subject’s diffusion images to the template space, which was needed in some cases, while no manual correction was applied to the results for the automatic registration performed in FSL pipeline. Although, the ALPS index calculated by using both DSI Studio pipeline and FSL pipeline demonstrated favorable inter-scanner reproducibility, inter-rater reliability, and test-retest repeatability, the ALPS index analyzed with FSL pipeline showed better agreement between raters and test-retest sessions, but slightly lower consistency across scanners when compared to DSI Studio pipeline. Which processing steps would have influence on the validation results require further investigation.

In the original DTI-ALPS paper by Taoka et al. [24], the authors proposed that the perivascular space run in the same direction as the medullary veins at the level of the lateral ventricle body and the association and projection fibers run orthogonal to the direction of perivascular space. Subsequent studies applied susceptibility weighted imaging (SWI) images to identify the medullary veins and converted the FA map and diffusivity maps into the SWI space, in order to define the location of ROIs in the association and projection fibers [30, 44-48]. This method needs to be evaluated in future validation studies. In our study, the placement of ROIs was only based on the identification of association and projection fibers according to the color-coded FA map or JHU-ICBM-DTI-81-white-matter Labeled Atlas, which is the major limitation of the present study.

In summary, the present study found that the ALPS index had favorable inter-scanner reproducibility, inter-rater reliability, and test-retest repeatability, offering a robust potential biomarker for evaluating GS clearance function in neurological disorders and in particular cSVD. Additionally, the present study provides two processing pipelines for DTI-ALPS calculation. Further validation of the two ALPS processing pipelines in multicenter clinical studies is warranted.

## Data Availability

The script for FSL pipeline can be downloaded at http://loft-lab.org/index-5.html.
